# King Edward's Hospital Fund for London

**Published:** 1909-12-18

**Authors:** 


### KING EDWARD'S HOSPITAL FUND FOR LONDON.

A meeting of the President and General Council of King Edward's Hospital Fund for London, for awarding grants to the hospitals, convalescent homes, and consumption sanatoria for the present year, was held on the 13th inst. at Marlborough House, the Prince of Wales being in the chair.

Those present were the Bishop of London, the Chief Rabbi (the Rev. Dr. Adler), Lord Bessborough, Lord Rothschild, Lord Revelstoke, Lord Farquhar, the Postmaster-General, the President of the Hospital Sunday and Hospital Saturday Funds (the Lord Mayor), the Chairman of the County Council (Sir Melvill Beachcroft), the Governor of the Bank of England (Mr. Reginald E. Johnston), the President of the Royal College of Surgeons (Mr. Henry T. Butlin), Sir Joseph Dimsdale, Sir Edgar Speyer, Sir Trevor Lawrence, Sir W. S. Church, Sir Julius Wernher, Lieutenant-Colonel Sir Arthur Bigge, Sir Henry Burdett, Sir Horace Marshall, Sir W. J. Collins, M.P., Sir John Craggs, Dr. Edwin Freshfield, Mr. John G. Griffiths, Professor W. Osler, Mr. Albert G. Sandeman, Mr. Hugh C. Smith.

The minutes of the last meeting having been read and confirmed, letters of regret were reported from the following: The Lord Lieutenant of the County of London (the Duke of Fife), the Duke of Norfolk, the Bishop of Southwark, Archbishop Bourne, Lord Iveagh, Lord Strathcona and Mount Royal, the Speaker of the House of Commons, the Master of Elibank, M.P., the President of the Royal College of Physicians (Sir R. Douglas Powell), Sir Savile Crossley, Sir Ernest Cassel, Mr. Thomas Burt, M.P., Mr. J. Danvers Power, Mr. Frederick M. Fry, Mr. William Latham, K.C., Mr. J. Pierpont Morgan, jun.

The following is the official report of the proceedings:

### Income and League of Mercy.

Lord Rothschild reported that the amount received by the fund to December 9, after payment of expenses, was £133,977 5s. 4d.

Sir Henry Burdett stated that although it had not been a good year for small contributions, and there had been various other difficulties, the League of Mercy would be able to renew its contribution of £19,000 to the fund. The amount distributed by the League to hospitals in the home counties had been increased to £1,980, thus making a total of practically £21,000. He thought this was a remarkably good result, and reflected great credit on all the workers of the League, and also on its new Secretary.

The Prince of Wales said: My Lords and Gentlemen,—You will all be delighted to hear what Sir Henry Burdett has said with regard to the League of Mercy. I know the extreme difficulty that there is in collecting these small sums, and I am sure you will agree that the results are most satisfactory.

### Report of Distribution Committee.

The Chairman of the Distribution Committee (Sir William Church) read the report of that committee as follows:—

1. The Council having this year placed the sum of £147,000 in the hands of the committee for distribution among the London hospitals, as against £135,000 in 1908, the committee beg to report as follows:—

2. The number of hospitals applying for grants is 103, as against 105 last year.

3. In the new building of the Royal National Orthopædic Hospital, opened by the King in July last, 130 out of the 200 beds for which the hospital is designed are already available for use. This represents a further stage in the amalgamation of the orthopædic hospitals. In addition to the grant for maintenance, the committee have recommended a substantial sum towards the reduction of the debt on the new building.

4. The amalgamation of the Hampstead and North-West London Hospitals is now complete. The out-patient department, with a few beds for emergencies, will be maintained in the poor district of Kentish Town, formerly served by the North-West London Hospital, while the in-patient department of the combined institutions is situated upon the higher ground of Hampstead. The committee, in addition to making a maintenance grant, have now set aside the sum of £3,000 on account of the grants promised in connection with the reconstruction of the out-patient department.

5. With regard to the Throat, Nose, and Ear Hospitals, no steps, so far as the committee are aware, have been taken during the year in the direction of amalgamation. The committee in their last report recommended that the sums set aside for this purpose in past years should not be held over indefinitely, and they consider that the time has now come to apply to these awards the rule that grants for special purposes should be reconsidered every two years if they remain unapplied. The committee, therefore, while setting aside the sum of £2,500 as the grant this year towards the amalgamation, recommend that the grants of £1,000 made in 1906 and £2,000 made in 1907 should now lapse.

6. The following grants made in 1907 for special purposes and retained in accordance with the rule also remain unapplied:

East London Hospital for Children, £618 16s. 6d., being the balance of a grant of £1,000 to improvements.

Miller General Hospital for South-East London, £289, being the balance of a grant of £1,250 to extension.

Royal Hospital for Diseases of the Chest, £1,000 granted to open a fund for a country sanatorium.

The committee recommend that the unpaid balances of the grants to the East London Hospital for Children and the Miller General Hospital should be renewed. With reference to the Royal Hospital for Diseases of the Chest, they are informed that the governing body of the hospital have not been able to see their way to undertake a scheme for the provision of a country sanatorium. In the circumstances they recommend that the grant should lapse, but they are glad to hear that the Convalescent Homes Committee of the Fund are making inquiries with a view to a scheme by which in certain existing sanatoria beds might be made available for patients from metropolitan hospitals.

7. In connection with the resolution of the General Council requesting hospitals which are contemplating extension or reconstruction to submit their proposals to the Fund, the committee have again had before them several schemes of greater or less magnitude. To some of these the committee have raised no objection, in some they have suggested modifications, while two or three are still under consideration. In two cases, however, the schemes appeared to be open to such serious objections on general grounds that the committee felt compelled to recommend that the approval of the Fund should be withheld.

In one case, that of the National Hospital for Diseases of the Heart, it was proposed to rebuild and enlarge the hospital at a cost of £25,000. It is admitted that the present building is unsuitable for the purposes of a hospital, but, considering the demands now made upon the charitable public for the maintenance and improvement of hospital accommodation in London, the committee could not recommend a scheme for the expenditure of a large sum of money in making special provision for cases which can be equally well treated elsewhere.

The other case raised the question of the proper method of supplying the need for a general hospital in Woolwich. In their last report the committee emphasised the policy adopted by the Fund of assisting in the provision of hospital accommodation in the outlying districts, and they consider that the special circumstances of Woolwich give that neighbourhood a strong claim upon the assistance of the Fund. The committee, however, saw grave objections to the particular scheme laid before them, which contemplated the gradual evolution of a hospital out of general wards connected with and subordinate to a small institution primarily designed for the training of midwives. The committee consider that the first step towards meeting the needs of Woolwich should be the formulation of a scheme for the provision of an independent general hospital of fair size, to which any other kindred objects should be subordinate, and they have announced their readiness, in the event of a representative movement in this direction, to afford substantial assistance.

8. In further connection with the provision of hospital accommodation in the outlying districts of London, the committee are of opinion that the needs of Wimbledon would be better served by one adequate hospital than by two independent institutions for which separate building schemes are proposed.

9. The sum of £5,000 voted towards the removal of King's College Hospital to South London increases the total amount contributed by the Fund to this object to £32,000.

10. The reports of the visitors, which every year prove of increasing value, have as heretofore received most careful consideration by the committee, and many of their observations have been brought to the notice of the managers of the hospitals concerned; nor have the committee failed to call attention to instances in which the statistical report affords evidence that considerably greater economy might be exercised without impairment of efficiency.

The details of the awards to the various hospitals are appended :—

### Grants to Hospitals.

The Distribution Committee desire to draw attention to the fact that the absence of a grant does not necessarily imply dissatisfaction.

Alexandra Hospital for Children.—£400.
Belgrave Hospital.—£1,000; to reduce debt, on the understanding that if no substantial improvement is effected within the next year in the financial position of the hospital the Fund will not make another grant.
Blackheath and Charlton Cottage Hospital.—£100.
Bolingbroke Hospital.—£1,600.
British Lying-in Hospital.—£300.
Cancer Hospital.—The committee are glad to see that no assistance from the Fund is required. The committee regret that the regulations of the revised uniform system of accounts are not observed.
Central London Ophthalmic Hospital.—£50.
Central London Throat and Ear Hospital.—See paragraph 5 of report.
Charing Cross Hospital.—£4,000; of which £1,500 to reduce debt.
Chelsea Hospital for Women.—£500.
City of London Hospital for Diseases of the Chest, Victoria Park.—£4,000. The committee trust that public support will be received to maintain the beds recently opened.
City of London Lying-in Hospital.—£1,000; of which £500 to reduce debt.
Clapham Maternity Hospital.—£200. The committee will be prepared to recommend substantial assistance towards an approved scheme of reconstruction.
Dreadnought Hospital.—£4,000; of which £1,000 to renewal of floors.
Ealing Cottage Hospital.—£1,000; to rebuilding scheme.
East End Mothers' Lying-in Home.—£1,000; of which £750 to reduce debt.
East London Hospital for Children.—£1,750.
Eltham and Mottingham Cottage Hospital.—£150; towards provision of casualty accommodation.
Evelina Hospital.—£150.
French Hospital.—£200.
Friedenham Hospital.—£50.
General Lying-in Hospital.—£300.
German Hospital.—£200.
Gordon Hospital for Fistula, etc.—£25.
Great Northern Central Hospital.—£5,000; of which £2,500 to reduce debt.
Grosvenor Hospital for Women.—£400; of which £50 to reduction of overdraft.
Guy's Hospital.—£9,000.
Hampstead General Hospital.—£3,000; of which £1,500 to reduce debt and £1,500 maintenance grant as agreed upon amalgamation with North-West London Hospital.
Home and Infirmary for Sick Children.—£200.
Home for Mothers and Babies, Woolwich.—£100; to improvements.
Hospital and Home for Incurable Children, Hampstead.—£25.
Hospital for Consumption, Brompton (including Sanatorium at Frimley).—£3,000.
Hospital for Diseases of the Throat.—See paragraph 5 of report.
Hospital for Epilepsy and Paralysis.—£750.
Hospital for Invalid Gentlewomen.—£500; to rebuilding.
Hospital for Sick Children.—£2,500; of which £1,000 to reduce debt on recent improvements.
Hospital for Women, Soho Square.—£1,000; towards purchase of adjacent site secured during the present year. The committee has extended the period for the collection of the balance of the sum required for the reconstruction scheme, laid down as a condition of last year's grant, from October 31, 1909, to March 31, 1910.
Hospital of St. John and St. Elizabeth.—£500; of which £150 to X-ray apparatus.
Infants' Hospital.—£25.
Italian Hospital.—£400.
Kensington and Fulham General Hospital (late Queen's Jubilee Hospital).
Kensington Dispensary and Children's Hospital.—£25.
King's College Hospital.—£6,250; of which £5,000 to removal fund.
Leyton, Walthamstow, and Wanstead Children's and General Hospital.—£100.
London Fever Hospital.
London Homœopathic Hospital.—£550.
London Hospital.—£12,000.
London Lock Hospital.—£3,500; of which £500 to maintenance of Harrow Road Hospital, and £3,000 to the rebuilding of the out-patient department in Dean Street on condition that the work is put in hand at once. The committee trust that the sum still needed for the nurses' home at Harrow Road will soon be subscribed by the public.
London Temperance Hospital.—£2,000; of which £1,000 to reduce debt.
London Throat Hospital.—See paragraph 5 of report.
Maternity Charity and District Nurses' Home, Plaistow.—£75.
Medical Mission Hospital, Balaam Street, Plaistow.—£350.
Memorial Hospital, Mildmay Park.—£50.
Metropolitan Hospital.—£5,000; of which £3,000 to structural improvements.
Middlesex Hospital.—£4,000.
Middlesex Hospital Cancer Charity.—The committee are glad to see that no assistance from the Fund is required this year.
Mildmay Mission Hospital.—£400; of which £50 to overdraft.
Miller General Hospital for South-East London.—£2,000; of which £1,500 to extension, subject to scheme being approved.
Mount Vernon Hospital for Consumption (including Sanatorium at Northwood).—£2,500; of which £1,000 to reduction of mortgage debt.
National Anti Vivisection Hospital.
National Dental Hospital.—£150.
National Hospital for Diseases of the Heart.
National Hospital for the Paralysed and Epileptic.—£1,500.

New Hospital for Women.—£500.
Norwood Cottage Hospital.—£100.
Paddington Green Children's Hospital.—£2,000; of which £1,250 to reconstruction of out-patient department, subject to approval of scheme.
Passmore Edwards Acton Cottage Hospital.—£50.
Passmore Edwards East Ham Hospital.—£25.
Passmore Edwards Hospital for Willesden.—£25.
Passmore Edwards Cottage Hospital, Wood Green.—£25.
Poplar Hospital.—£500.
Prince of Wales's General Hospital.—£4,000; of which £1,500 to reduce debt.
Queen Charlotte's Lying-in Hospital.—£750.
Queen's Hospital for Children.—£2,750; of which £1,000 to reduce debt.
Royal Dental Hospital of London.—£500.
Royal Ear Hospital.—See paragraph 5 of report.
Royal Eye Hospital.—£750.
Royal Free Hospital.—£4,000.
Royal Hospital for Diseases of the Chest, City Road.—£1,000.
Royal Hospital, Richmond.—£250; of which £150 to improvements.
Royal London Ophthalmic Hospital.—£2,500.
Royal National Orthopædic Hospital—£6,500; of which £5,000 to reduce building debt.
Royal Waterloo Hospital for Children and Women.—£1,500.
St. George's Hospital.—£1,500.
St. John's Hospital for Diseases of the Skin.—£500; of which £400 to reduce building debt.
St. John's Hospital, Lewisham.—£250; of which £50 to recent improvements.
St. Luke's House.—£25.
St. Mark's Hospital.—£200; of which £100 to improvements in nursing accommodation.
St. Mary's Hospital.—£5,000; of which £1,000 to reduce debt.
St. Mary's Hospital for Women and Children, Plaistow.—£1,500; of which £1,000 to building.
St. Monica's Hospital.—£50.
St. Peter's Hospital for Stone.—£150.
St. Saviour's Hospital, Osnaburgh Street—£200.
St. Thomas's Hospital.
Samaritan Free Hospital.—£1,000.
Santa Claus Home.—£25.
University College Hospital.—£4,250.
Victoria Hospital for Children.—£1,500; of which £300 to reduce building fund deficit.
West End Hospital for Diseases of the Nervous System.—£250.
Western Ophthalmic Hospital.
West Ham and East London Hospital.—£2,000; of which £1,500 to building.
West London Hospital.—£3,000; of which £1,000 to reduce maintenance debt.
Westminster Hospital.—£3,500; of which £500 to reduce debt.
Wimbledon Cottage Hospital.—£50.—Total £141,500

### SPECIAL GRANTS.

Set aside for amalgamation of throat and ear hospitals.—£2,500.
Set side for amalgamation of Hampstead and North-West London Hospitals.—£3,000.—Total £5,500.

### SUMMARY.

| | |
|---|---|
| Grants ... ... ... ... ... ... ... | £141,500 |
| Special grants set aside for amalgamations ... | 5,500 |
| Total grants to hospitals for the year 1909 ... | 147,000 |

### CONVALESCENT HOMES REPORT.

In the absence of the Chairman of the Convalescent Homes Committee (Mr. William Latham, K.C.), the Secretary read the report of that committee as follows :—

1. The Council have this year placed the sum of £3,000 in the hands of the committee for distribution among convalescent homes and consumption sanatoria. This makes, with the sum of £1,000 set aside last year for consumption sanatoria but not allocated, a total distribution of £4,000, as against £5,000 provided for in 1908, of which only £4,000 was actually distributed.

2. The number of applications eligible for consideration amounted to thirty-seven from convalescent homes and four from consumption sanatoria, as against thirty-three and five respectively last year.

3. The committee have received new applications from several convalescent homes, to most of which grants are recommended in consideration of the number of London patients admitted during the past year. At the same time, the committee have adhered to some extent to their original policy of giving special consideration to the convalescent homes closely connected with London hospitals.

4. The committee regret again to have to report the smallness of the number of applications from consumption sanatoria, and the very limited opportunities which have hitherto presented themselves for assisting by means of grants in the provision of additional beds for the immediate accommodation of those patients in London hospitals who urgently need country treatment.

5. The committee, however, have under consideration proposals for securing for patients in London hospitals the right of admission to a certain number of beds at a sanatorium now in course of erection. Should this sanatorium upon inspection by the Fund's visitors be found to be eligible for a grant on this basis, the committee hope to be in a position to make a recommendation next year. They trust that this scheme, if successful, may lead to the conclusion of similar arrangements elsewhere, and that this possibility may be borne in mind by the promoters of sanatoria within reach of London. In cases, for instance, where a larger number of beds could be opened at a sanatorium without proportionate additional expenditure upon the administrative sections or increase in the fixed charges, such an arrangement might well result in greater economy in the management of the sanatorium, besides saving the extra cost of erecting and maintaining separate establishments for London patients.

6. The committee would again express their indebtedness to the visitors who undertook the inspection of consumption sanatoria in the country, and whose reports have been of the greatest value.

The grants recommended to the various institutions are appended :

### A.—CONSUMPTION SANATORIA.

The Convalescent Homes Committee desire to draw attention to the fact that the absence of a grant does not necessarily imply dissatisfaction.
The address of the institution follows the title, and the committee's remarks, where any are made, follow the amount of the grant:—
Children's Sanatorium, Holt, Norfolk.—£500; to the building fund for a permanent sanatorium, subject to the scheme being approved.
Eversfield Chest Hospital, St. Leonards.
Kelling Sanatorium, Holt, Norfolk.—£50.
National Association for the Establishment and Maintenance of Sanatoria for Workers, Benenden, Kent.—£500; to reduce debt.
Total grants recommended.—£1,050.

### B.—CONVALESCENT HOMES.

Alexandra Hospital for Children, Painswick.—£25.
All Saints' Convalescent Home for Children, Highgate.—£25.
Baldwin Brown Home for Convalescent Poor, Herne Bay.—£25.
Charing Cross Hospital, Limpsfield.—£225; of which £150 to reduce debt; £75 maintenance grant with a view to encouraging the admission without payment of patients direct from the hospital.
Chelsea Hospital for Women, St. Leonards.—£50; with a view to encouraging the admission without payment of patients direct from the hospital.
Convalescent Police Seaside Home, Hove.—£25.
East London Hospital for Children, Bognor.—£250.
Home and Infirmary for Sick Children, Sydenham (Broadstairs).—£25.
Hospital for Sick Children, Great Ormond Street (Highgate).—£250.
King's College Hospital (Hemel Hempstead).—£100.
London Hospital (Tankerton).—£100.
Mary Wardell Convalescent Home for Scarlet Fever (Stanmore).—£25.
Metropolitan Hospital (Cranbrook).—£50.
Middlesex Hospital (Clacton).—£500.
Mildmay Mission Hospital (Hendon).—£100.

Mrs. Gladstone's Free Convalescent Home (Mitcham).—£100.

Mrs. Kitto's Free Convalescent Home (Reigate).—£75.

National Hospital for the Paralysed (Finchley).—£100; with a view to facilitating the admission of patients without payment.

Paddington Green Children's Hospital (Slough).—£50.

Poplar Hospital (Walton-on-Naze).—£50.

Queen Charlotte's Lying-in Hospital (Kilburn).—£50.

St. Andrew's Convalescent Home (Folkestone).—£100.

St. Mary's Home for Children (Broadstairs).—£50.

St. Michael's Home for Men and Women (Westgate-on-Sea).—£25.

Sunshine Home of Recovery (Hurstpierpoint).—£50; to reduce debt.

Surgical Home for Boys (Banstead).—£50; of which £25 to mortgage redemption fund.

Victoria Home for Invalid Children (Margate).—£200; to building.

Victoria Hospital for Children, Chelsea (Broadstairs).—£250.

Winifred House Invalid Children's Convalescent Hospital Home (Tollington Park).—£25.

*Summary.*

| | |
|---|---|
| Grants to Consumption Sanatoria ... ... ... | 1,050 |
| Grants to Convalescent Homes ... ... ... ... | 2,950 |
| Total grants recommended (including distribution of grant of £1,000 to consumption sanatoria reserved in 1908) ... ... ... ... ... ... ... | £4,000 |

### The Prince's Speech.

The Prince of Wales, in moving the adoption of the reports and awards, said: My Lords and gentlemen,—The interesting reports of the Distribution and Convalescent Homes Committees, to which we have listened, will, I hope, be considered satisfactory, and I have much pleasure in moving their adoption. The largest donation received during the year was one of £4,775, representing one-half of the present available surplus of the Franco-British Exhibition. Our best thanks are due to the Secretary of State for Foreign Affairs, in whose hands the disposal of this sum was left, for having given it to the Fund. (Hear, hear.) The legacies this year have not equalled the exceptional amounts received in 1907 and 1908. But by following the practice adopted in those years of distributing a proportion based approximately on a five years' average of the amount received from this source, it has been possible, with due regard to the future, to distribute the sum of £150,000 this year. This amount has more than once been publicly referred to as our aim. It was mentioned in the King's letter inaugurating the Fund, and was alluded to by Lord Mount Stephen when he made his last munificent donation of £100,000 to capital. Its attainment, therefore, represents, like the distribution of £50,000 in 1900, and £100,000 in 1903, a landmark in the history of the Fund. (Hear, hear.) It is my pleasing duty to convey to you from the King the expression of his gratification at this achievement, upon which his Majesty offers us his hearty congratulations. But it does not follow that a distribution of £150,000 can be maintained without effort. The income from investments is not yet £70,000, and for the balance we have to rely upon annual subscriptions, donations, including the contributions of the League of Mercy, and legacies. I am sorry to find that there has been a diminution in the annual subscriptions. It is to be hoped that this may only be a temporary loss, and that with a revival of trade there may be a corresponding increase in our income obtained from this source.

The reports of the Distribution and Convalescent Homes Committees give evidence, as usual, of the care with which these committees do their work. It must not be forgotten that while the awards are made in respect of work done in the current year the financial statistics upon which they are based must necessarily be derived from the audited accounts of the previous year. The study of these accounts show that while the year 1908 did not prove to be so bad as anticipated, the demands on the Fund were considerably greater than in 1907. In three or four cases you will notice that the Distribution Committee recommends that the Fund should make specially large grants to hospitals which were in pecuniary difficulties and were hampered by debt. These grants are not recommended without most careful inquiry. I think that the power of assisting institutions in exceptional difficulties, after such inquiries, is of the greatest benefit to the hospitals generally. The report of the Distribution Committee calls attention to two successful amalgamations—that of the Orthopædic Hospitals and that of the Hampstead and North-West London Hospitals; and I am sure that the Council will be right in making substantial grants to these amalgamated institutions. I regret that the hope expressed last year of similar action on the part of the throat, nose, and ear hospitals has not yet been fulfilled. The Distribution Committee's recommendation for this year, if confirmed, will slightly diminish the total amount set aside for this amalgamation. That amount, I think, still exceeds the individual grants which would probably have been made to the separate hospitals.

It will be noticed that the consideration of proposals for the extension or improvement of hospital accommodation has again occupied the attention of the Distribution Committee. These schemes arise from the necessity for hospitals to keep pace with the improvements in medical science; and from the growth or shifting of population both in the centre and in the suburbs. The examination of them has added greatly to the labours of the committee. After reading the minutes of each meeting, I can bear witness to the time and trouble expended on this work, no scheme of any magnitude being passed without a visit and report by members of the committee specially deputed. The work of the Convalescent Homes Committee, which for many years was limited to the distribution of the generous donation of £1,000 from the London Parochial Charities, is now extended to dealing with the amount voted by the Council for convalescent homes and consumption sanatoria. Considering the enormous demand for accommodation in the country for consumption patients from the London hospitals, it is much to be regretted that the possible openings in this direction are at present so few. I trust that the managers of existing or of future sanatoria within reach of London will appreciate the readiness of the committee to enter into negotiations to secure beds for the accommodation of patients from metropolitan hospitals. (Hear, hear.)

The Executive Committee have this year had under consideration the question of the expenses of charity entertainments. On their recommendation I appointed a special sub-committee, which, under the chairmanship of Lord Richard Cavendish, collected evidence on the subject and presented a report published last August. The conclusion arrived at was that, while there was no evidence of widespread extravagance or abuse, there were certain dangers to be avoided, and these the report dealt with in detail. It is by no means easy to suggest rules to enable hospitals to protect their interests without hampering the legitimate and valuable efforts of their friends. The Executive Committee, I think wisely, decided not to lay down definite regulations, but to circulate the report, thus hoping to assist the hospitals in framing their own regulations, and to elicit their views as to further action by the Fund in the matter.

You will remember that last year the appointment of the proposed committee of inquiry into the out-patient question was postponed pending the issue of the report of the Poor Law Commission. Much of the evidence taken by that Commission on the question of medical relief is now available, and I trust that it may be possible to appoint the committee in the coming year. It is to be hoped that before that time Mr. Fry, whose absence to-day we all regret, will be fully restored to health. His illness, originally resulting from an accident incurred while travelling to London on the business of the Fund, has, I fear, been aggravated by the work connected with the meetings of the Distribution Committee.

And now, in the name of the Council, I ask the honorary officials and members of the League of Mercy to accept our hearty thanks for their strenuous and most successful efforts, by which the Fund has benefited to the amount of £19,000 this year. To the honorary officials of the Fund itself we owe a special debt of gratitude. The yearly increase of their work only seems to call forth from them redoubled zeal and readiness to cope with it. To Mr. Maynard, our efficient and hard-working secretary, I offer our best thanks. We have sustained great loss in the deaths of Mr. Clutton and Mr. Marcus Gunn, both eminent in their profession, who had for many years rendered invaluable service to the Fund as members of our Board of Visitors. I now have the pleasure of moving the adoption of reports of the Distribution and Convalescent Homes Committees. (Hear, hear.)

The Bishop of London, in seconding the adoption of the reports, said that he was certain that he would be expressing the opinion of all the Council if he first offered his Royal Highness their heartiest congratulations on the magnificent result that had been achieved by the Fund in distributing the sum of £150,000. He thought that result was largely owing to the personal interest that his Royal Highness had always taken in the Fund. He would only mention two points. Being constantly in the London hospitals, his work took him about from one hospital to another, and it was quite impossible to over-estimate the encouragement which these grants gave to the struggling institutions, and the way in which they drew forth subscriptions. But apart from stimulating other subscriptions, the Council was filling a very long desired place as a central board for improving hospital management, and he had himself found over and over again improvements that he wished to see put into force accomplished by means of the Fund. He felt quite certain that the Council under his Royal Highness's presidency was increasing the confidence of the charitable public in these institutions altogether, and he had very great pleasure in seconding the adoption of the reports.

The adoption of the reports was then put and carried unanimously.

On the motion of the Chairman of the London County Council (Sir Melvill Beachcroft), seconded by Lord Revelstoke, it was resolved that the cheques for the grants should be posted on December 16.

On the motion of Earl of Bessborough, seconded by the President of the Royal College of Surgeons (Mr. Henry Butlin), a recommendation of the Executive Committee on the subject of rules and regulations was adopted.

### Changes and Appointments.

The President then said: My Lords and Gentlemen,—I intend to appoint the same members of the General Council who have served this year to serve again, with the addition of the following: Lord Sandhurst, Canon Barnett, and Sir Cooper Perry. With regard to the committees, I intend, later on, to reappoint the present members of these with very few changes. I regret that we shall lose the services of three gentlemen who are unable to continue to serve—Sir Trevor Lawrence, who has given us the best of his time for many years past on the Distribution Committee, and Lord Richard Cavendish and Canon Barnett, who, though only recently appointed, have been useful members on the Executive and Distribution Committees respectively. We now have to consider the resolution delegating powers to the Finance, Distribution, and Convalescent Homes Committees. The delegation resolutions as amended last year have worked well, and if the Council is willing to reaffirm them now the proceedings at the meeting in the new year need only be formal. With reference to the Executive Committee it is necessary to add a clause empowering the Committee to provide for the expenses of the out-patient inquiry.

On the motion of Lord Farquhar, seconded by Sir Edgar Speyer, a resolution was passed delegating powers to the Finance, Distribution, and Convalescent Homes Committees.

On the motion of Sir Joseph Dimsdale, seconded by the Governor of the Bank of England, a resolution was passed delegating powers to the Executive Committee.

His Royal Highness then moved:—

"That the keys of the lock under which the corporate seal is kept be held as follows: One of the three keys comprising one triplicate set shall be held respectively by the Earl of Bessborough, the Lord Rothschild, and Mr. Frederick M. Fry, and one of the three keys comprising the other triplicate set shall be held respectively by Sir Savile Crossley, Mr. John G. Griffiths, and Mr. Hugh Colin Smith."

The motion was seconded by the Chief Rabbi and carried.

### Thanks to the Prince.

The Lord Mayor (Sir John Knill), in moving a vote of thanks to his Royal Highness for presiding, said he had the great pleasure and the great honour of proposing a vote of thanks to his Royal Highness for presiding at the meeting that day. Knowing the keen interest his Royal Highness took in the Fund, he would ask the Council to join with him in giving thanks.

Sir William Collins said he had great pleasure in seconding the resolution and in thanking his Royal Highness for the gracious and encouraging words which had fallen from him that day. Also, as an honorary officer of the League of Mercy, he would like to thank his Royal Highness for the handsome encomium he had passed on the work of the League. That work was carried out now in a larger number of districts than before, and during the ten or eleven years in which it had been at work the League had handed over to the King's Hospital Fund the sum of £135,000. Only those who worked as honorary officers of the Fund and of the League of Mercy knew how incalculable the personal interest taken by his Royal Highness, and by other members of the Royal Family, in this work was in maintaining and consolidating it, and on behalf of the Council and of the League he had the greatest pleasure in seconding the vote of thanks to his Royal Highness. (Hear, hear.)

The vote was carried by acclamation.

The Prince of Wales said: My Lords and Gentlemen,—I beg to thank the Lord Mayor and Sir William Collins for the kind words they have used in proposing and seconding the vote of thanks to me. It is always a great pleasure to me to have this meeting held in my house, and especially so when we can all congratulate ourselves upon the very prosperous state of the Fund.

The proceedings then terminated.

